# Immune cell residency in the nasal mucosa may partially explain respiratory disease severity across the age range

**DOI:** 10.1038/s41598-021-95532-3

**Published:** 2021-08-05

**Authors:** Konner Winkley, Dithi Banerjee, Todd Bradley, Boryana Koseva, Warren A. Cheung, Rangaraj Selvarangan, Tomi Pastinen, Elin Grundberg

**Affiliations:** 1 Genomic Medicine Center, Children’s Mercy Research Institute, Children’s Mercy Kansas City, Kansas City, MO 64108 USA; 2 Department of Pathology and Laboratory Medicine, Children’s Mercy Kansas City, Kansas City, MO 64108 USA

**Keywords:** Functional genomics, Mucosal immunology

## Abstract

Previous studies focusing on the age disparity in COVID-19 severity have suggested that younger individuals mount a more robust innate immune response in the nasal mucosa after infection with SARS-CoV-2. However, it is unclear if this reflects increased immune activation or increased immune residence in the nasal mucosa. We hypothesized that immune residency in the nasal mucosa of healthy individuals may differ across the age range. We applied single-cell RNA-sequencing and measured the cellular composition and transcriptional profile of the nasal mucosa in 35 SARS-CoV-2 negative children and adults, ranging in age from 4 months to 65 years. We analyzed in total of ~ 30,000 immune and epithelial cells and found that age and immune cell proportion in the nasal mucosa are inversely correlated, with little evidence for structural changes in the transcriptional state of a given cell type across the age range. Orthogonal validation by epigenome sequencing indicate that it is especially cells of the innate immune system that underlie the age-association. Additionally, we characterize the predominate immune cell type in the nasal mucosa: a resident T cell like population with potent antiviral properties. These results demonstrate fundamental changes in the immune cell makeup of the uninfected nasal mucosa over the lifespan. The resource we generate here is an asset for future studies focusing on respiratory infection and immunization strategies.

## Introduction

Disease severity of respiratory virus infection tends to correlate with age. For example, influenza causes the most severe disease in the youngest and oldest populations^1,2^, while respiratory syncytial virus causes severe disease in very young children nearly exclusively^3^. However, in contrast to these viruses, SARS-CoV-2 which causes coronavirus disease of 2019 (COVID-19) is associated with significant higher numbers of mild and asymptomatic cases in children vs. adults^4^. While many hypotheses have been proposed to account for this discrepancy in age predilection of severe disease for SARS-CoV-2^5,6^, a recent study demonstrated that the nasal mucosa, the site of viral exposure, of children and adults infected with SARS-CoV-2 is different in terms of the early immune response as measured by bulk RNA sequencing^7^. This study found that children who have a similar viral burden to a cohort of adults had a transcriptional profile indicative of increased innate immune response with specific increases in Interferon, IL-1, IL-17, and NLRP3 signaling^7^. This important insight offers information that may help explain the differences in disease severity between young and old populations, however open questions remain. It is currently unclear if this increase in innate immune signaling is caused by differences in the immune cell population size between pediatric and adult groups, or if a similarly sized population of immune cells is simply more or less activated in the two age groups. Additionally, if differences in immune cell population sizes exist between pediatric and adult groups, it would be important to know if these disparities exist prior to infection or if they are the result of an increase in infiltration and proliferation of circulating and nasal mucosa resident immune cells after infection occurs.

We hypothesized that the nasal mucosal resident immune cell populations of pediatric and adult cohorts may be fundamentally different in uninfected conditions. We reasoned that profiling the nasal mucosa of uninfected individuals with greater age-resolution across the lifespan using single-cell RNA-sequencing (scRNAseq) rather than bulk RNA-sequencing, would provide insight into mechanisms that lead to increased innate immune response in the nasal mucosa of younger populations. To this end, we isolated and collected cells scavenged from clinical mid-turbinate swabs of SARS-CoV-2 negative individuals across the age range and profiled the nasal mucosa at single-cell resolution using the high-throughput 10X Genomics scRNAseq platform. We found that in uninfected conditions, the resident immune cell population of the nasal mucosa decreases dramatically with age. We demonstrate that cell types are transcriptionally similar across the age range with no signs of differential activation that correlates with age. Finally, we take advantage of the increased coverage of the resident immune cell types in our younger samples and provided a deep characterization of a major immune cell type present in the nasal mucosa with potential antiviral properties.

## Results

### Single-cell profiling of nasal mucosa across the life span

We characterized the composition of the nasal mucosa (NM) across the lifespan, by taking advantage of readily available clinical samples of NM-derived cells from 35 COVID-19-negative pediatric patients and healthcare workers. Specifically, we developed a protocol to isolate viable cells from salvaged respiratory specimens collected by mid-turbinate swabs shortly after completion of clinical testing. Then, we used a specimen pooling approach, where isolated cells from four to nine deidentified individuals of similar age groups were combined into a common pool (Supplemental Table 1). Applying scRNAseq, we captured and performed transcriptomic profiling in a total of 26,014 high quality cells across six age-distinctive pools covering the vast majority of the lifespan (average age per pool ranged between 1 and 50 years, Supplemental Table 1).

We used a genetic demultiplexing technique that does not require a priori knowledge of input genotypes to demultiplex these pools^8^. While we found that there was interindividual heterogeneity in cell type composition, no cell type in a pool originated completely from a single individual (Supplemental Fig. 1). Because inferences made on pooled population-level samples rather than individual level samples averages out the effects of individual heterogeneity and increases coverage of cell types in the age-specific pools, the following analyses were measured at the level of the population pools rather than the level of the individual.

**Figure 1:**
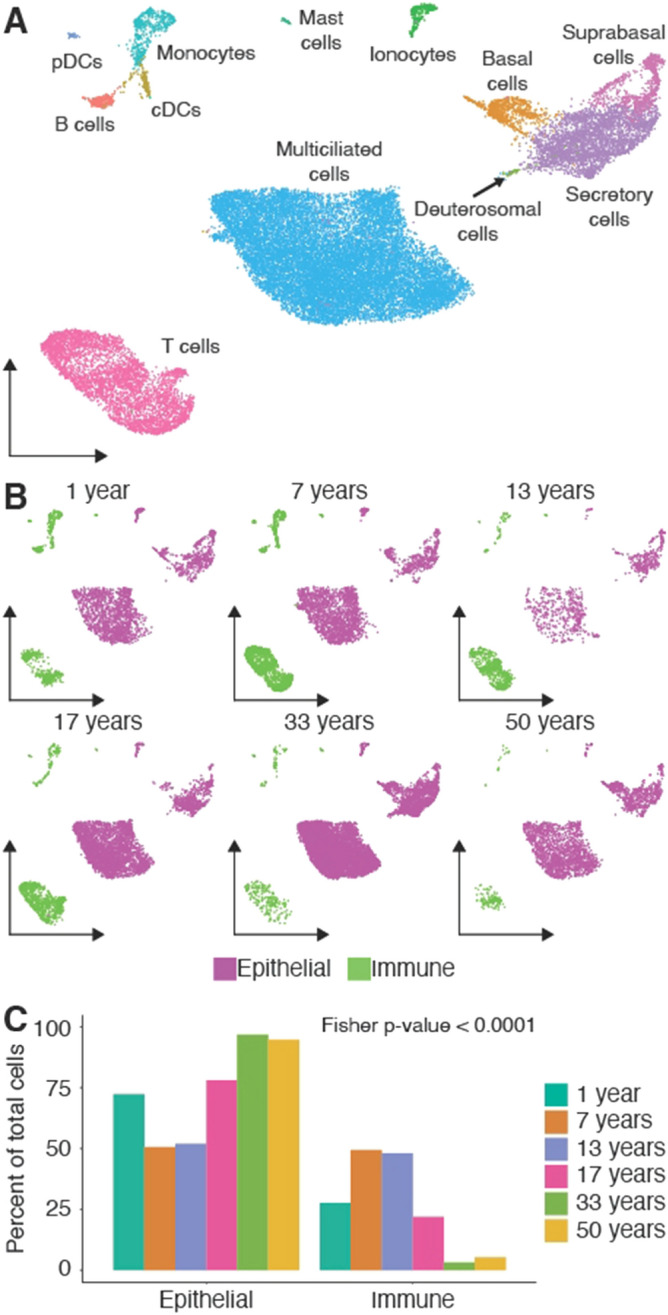
Immune cell residency decreases with age in the nasal mucosa. (**A**) UMAP projection of cells from nasopharyngeal swabs collected across the lifespan pools. Colors correspond to different cell types. (**B**) Same UMAP projection from A split by the 6 pools that make up the dataset. Cells are colored by a coarse cell type assignment where magenta cells are epithelial, and green cells are immune. (**C**) Bar plot quantification of B. *p*-values is for a Fisher’s exact test of association between age and cell type composition.

The transcriptomic profiles were used to group the ~ 26,000 cells into 12 distinct cell clusters and included all expected epithelial and immune cell types (Fig. 1A). Specifically, the epithelial cell types included multiciliated cells^9^, deuterosomal precursor cells^10,11^, secretory cells^9,12^, basal cells and suprabasal cells^12,13^. We also classified six distinct immune cell types which were: monocytes, conventional dendritic cells (cDCs), plasmacystoid dendritic cells (pDCs), B cells, Mast cells, and T cells. Additionally, we were able to identify *CFTR* expressing ionocytes^14^. We confirmed our cell type assignments by comparing marker genes for each cell type to previously published cell type markers of nasal epithelial cells^10,15,16^ (Supplemental Table 2).

### Age-association of cell type abundance in nasal mucosa

We next compared the proportions of cell types across the lifespan and found a striking negative relationship (Fisher test for interaction between age and coarse cell type proportion *p*-value < 0.0001) between age and immune cell residence in the NM samples (Fig. 1B,C). Within the immune cell types, we noted an age-associated decrease of multiple cell types, the most striking of which was monocytes (Supplemental Fig. 2A). This decline in cell abundance with age was not seen for the epithelial cell populations (Supplemental Fig. 2B). As some of the pools included skewed representation of female vs male samples, we performed a sub-analysis of female samples. Specifically, we took advantage of the genetic demultiplexing again by selecting cells only originating from females. After restricting our dataset within sex, we still observed a significant relationship between age and immune residence (Fisher test for interaction between age and coarse cell type proportion *p*-value < 0.0005) (Supplemental Fig. 3, indicating sex related differences are not confounding our results.

To validate the observed age-association of immune cell residency we performed several additional follow-up analysis: First, we generated similar scRNAseq data of independent collected samples using the same collection and pooling approach from infants whose average age was 4 months, further expanding the age-range in our analysis. We found high concordance in the proportions of cell types with our discovery pools derived from children (Supplementary Fig. 4). Next, we compared the cellular composition in our 33-year-old pool with a published dataset of healthy individuals whose average age was 36 years^16^. We again found high concordance in the proportions of cell types in this age-matched comparison (Supplemental Fig. 5). Finally, we performed orthogonal validation by whole-genome bisulphite sequencing of a set of independent NM samples from COVID-19 negative children and adults spanning a similar age range as our discovery samples (average age per pool ranged between 1 and 45 years) and using a similar pooling approach across five age-specific pools. Each pool was sequenced at high depth (~ 27 × unique read coverage, Supplementary Table 3) identifying methylation profiles on average 19 million CpGs per pool, each at > 10X of which 8.9 million CpGs were covered across all samples. We annotated the CpG profiles from each pool based on cell-specific regulatory elements and found that regulatory regions of the genome associated with immune cell activity^17^ were significantly less methylated (i.e. hypomethylated) in samples from younger individuals (Supplemental Fig. 6) suggesting higher transcriptional activity of immune cells in these samples. While the methylation levels were significantly (*p* < 0.0001) lower across the age range in both myeloid and lymphoid lineages, the changes were more pronounced in the myeloid lineage in line with our observation of the decline in monocytes (Supplemental Fig. 6). This further indicates the link between the innate immune system and the observed age-association of NM cell composition.

**Figure 2:**
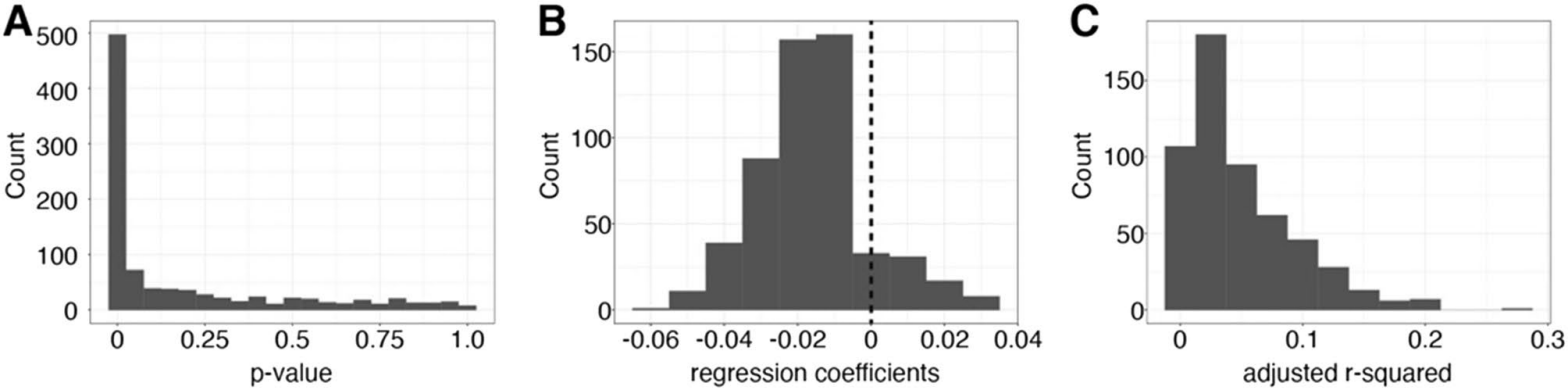
Transcriptional profiles of immune cells remain consistent across age groups. (**A**) Histogram of the distribution of *p*-values obtained from linear regression tests between age and expression level for all enriched markers of immune cell types. Bin with is 0.05. (**B**) Histogram of the distribution of beta-coefficients for the effect of age on expression level for all enriched markers of immune cell types that had a significant relationship between expression level and age. (**C**) Histogram of the distribution of adjusted r-squared value for the percent of expression variation explained by age for all enriched markers of immune cell types that had a significant relationship between expression level and age.

To identify if the immune residency in the nasal mucosa was qualitatively, as well as quantitatively, different across the age range, we explored genome wide expression differences in the NM immune cells with respect to age in our discovery cohort. We selected all marker genes for the immune cells that were at least log2 fold enriched (N = 950 cell type marker genes, 643 unique genes). We then tested for a linear association between expression of these marker genes and age within the selected cell type. Around half (N = 545 observations, 430 unique genes) of the marker genes were statistically significantly linearly related with age (*p* < 0.05) (Fig. 2A) (Supplemental Table 4). While most of the significant associations showed a decline in cell type specific expression with age (Fig. 2B), the magnitude of the association was often quite small (Fig. 2C). Overall, this result points to their being little evidence to support the hypothesis that cell types are transcriptionally different with respect to age and conclude that in uninfected conditions, the resident NM cells differ primarily in population proportion.

### Characterization of resident T-cells in nasal mucosa

**Figure 3:**
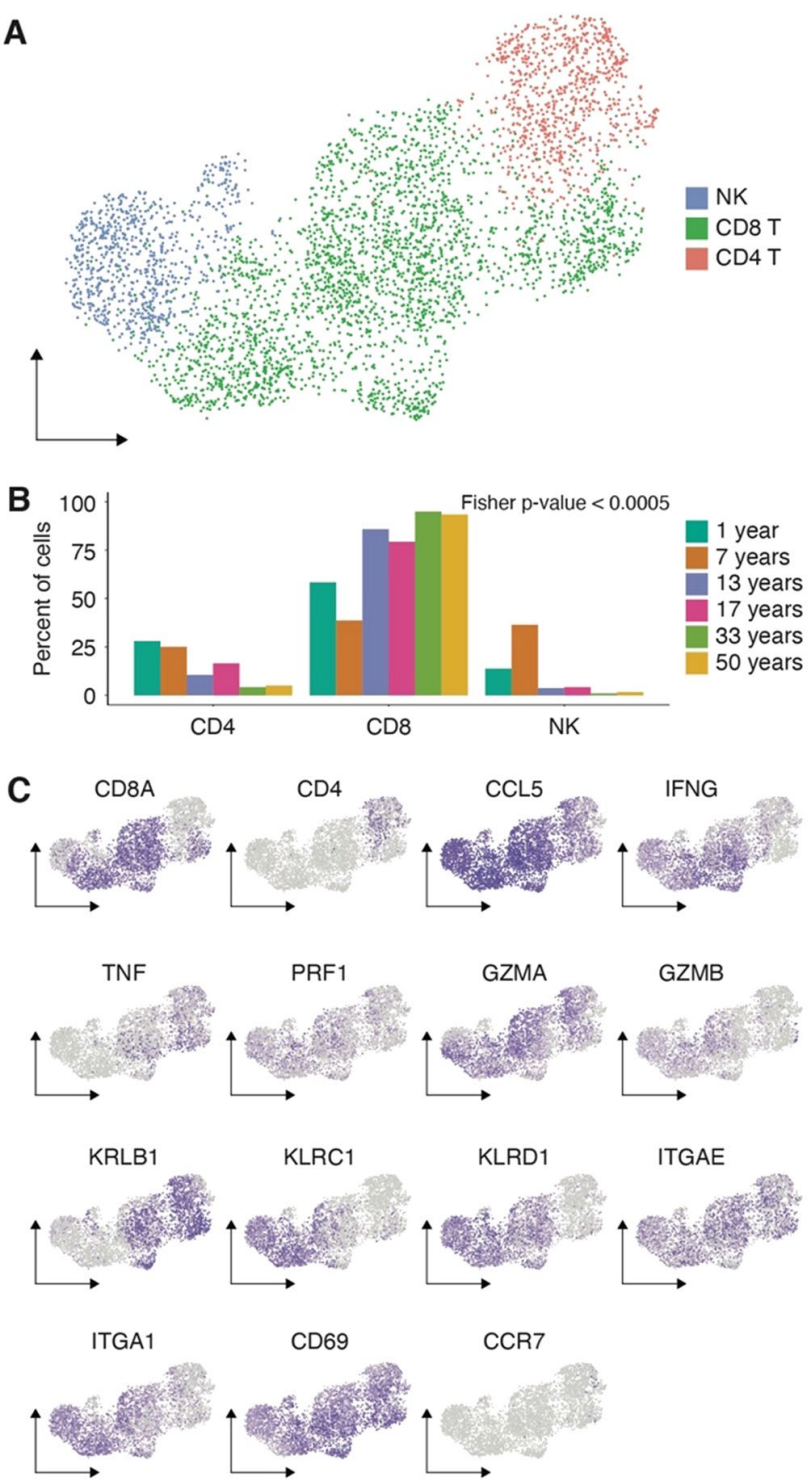
The primary immune cell population in the NM has an anti-viral resident-memory-like T cell transcriptional profile. (**A**) UMAP of the sub-clustering results for the T cell population. (**B**) Quantification of the proportion of each type of T lymphocyte present in across the age range. Bars represent percent of total T-lymphocytes in each pool that belong to each subtype. (**C**) Expression plots for selected markers of T cell subsets. UMAP projection is identical to A.

The predominant immune cell type in the NM samples studied here was a population of T cells comprising > 50% of the immune cells in each age-specific pool (Supplemental Fig. 2A). Many studies investigating the healthy NM with single-cell transcriptomics have only focused on adult samples^15,16,18^. As shown here, adults have a relatively small percentage of immune cells comprising their NM (Fig. 1C), this T cell population has typically lacked the coverage and resolution required for proper characterization. However, because of the increased immune cell residency in adolescents, we were able to capture and identify over 4,500 T cells in our dataset. To increase the resolution and to facilitate the classification of these cell types we subset and re-clustered the T cells, which were identified as CD4, CD8 and NK cells (Fig. 3A). We observed decreases in the proportion of CD4 and NK populations that make up the total T-like lymphocytes across the age range in line with our earlier results demonstrating the loss of innate immune system function with age (Fig. 3B). With this increased resolution, we found that these NM T cells were primarily CD8 positive and expressed genes encoding CCL5 and interferon gamma, TNF, PRF, granzymes, and killer-like receptors that are important for T cell effector function and antiviral activity. Additionally, these cells are strongly enriched for expression of tissue residency markers such as *ITGAE* (CD103), *ITGA1* (CD49a) and *CD69*, and are void of expression of *CCR7*, which is necessary for lymphocyte homing (Fig. 3C).

## Discussion

We present the first-of-its kind single-cell atlas of human nasal samples that spans the lifespan with important and novel coverage during childhood. Using this resource, we provide important insight into the immunobiology of the respiratory system during aging with implications for understanding the pathogenies of respiratory infections. Specifically, we find a clear pattern of decline in nasal resident immune cells with age that may partially explain observed age-associations of severity of respiratory diseases such as COVID-19 where children appear less affected and less prone to severe disease^19^ and orthogonally validate this finding using methylation profiles. The nasal epithelium is the portal for SARS-CoV-2 initial infection and transmission of the virus and the cellular response in this tissue is critical for determining the immune defense mechanism and clinical outcome after exposure. Yet, most studies have focused on blood or other organs in severely ill COVID-19 patients^16,20–22^. However, a recent study used NM samples and showed that early response to SARS-CoV-2 infection in children is marked by increased immune activation compared to infected adults where children expressed higher levels of genes associated with immune cells versus adults^7^. Here, we confirm this pattern but also expand the finding by demonstrating that age-related differences in the cellular structure of the NM exist even prior to infection.

The resolution of our single-cell NM atlas also allowed us to not only identify the immune residency trend with age but also to characterize the immune cell population in further detail. Specifically, we identified the primary T cell population in the NM as having a transcriptional profile very similar to that of resident-memory T lymphocytes (T_rm_) identified in non-lymphoid tissues and secondary lymphoid organs^23^. T_rm_ cells are known to occupy other tissues including the lungs in humans; they are important for local immune response there and can serve as a functional link between the innate and adaptative immune system^24–27^. While T_rm_ occupancy in the NM has yet to be described in humans, T_rm_ are known to occupy the NM in mice and help to prevent pulmonary dissemination of influenza virus^28^. This characterization of NM T cells demonstrates the potential insights gleaned by profiling NM samples across the lifespan. However, of note is the heterogeneous expression of some marker genes of T_rm_ fate within our identified T-cell population, and other key characteristics beyond transcripts detected are often used to distinctly categorize T cell sub populations. Therefore, this single-cell atlas can function as a recourse for hypothesis generation to other studies that may functionally characterize NM T cells.

The implications of this increased NM immune cell residency in younger individuals along with the decline across the lifespan will require further testing to fully explore. While our results provide a plausible explanation for the observed increase in innate immune signaling in pediatric populations compared to adults infected with SARS-CoV-2, they do not exclude the possibility that pediatric NM resident immune cells experience a higher level of activation upon infection independent of the resident immune cell proportion. Additional experiments to identify if this trend of decreased immune cell residency is a unique characteristic of the NM or if it is observed in other non-lymphoid tissues, along with identification of the biological mechanisms responsible for the decrease in residence will provide a more comprehensive picture to the process of aging and immune development. There also remains a question of how this increased immune cell residency may interact with disease severity of other respiratory viruses such as influenza, which severely affect younger individuals along with older individuals. Further, it is unclear how changes in immune cell residence in the NM during resting conditions may affect the levels of immune cells that infiltrate the NM upon viral infection. Finally, there remains the possibility that the observed decrease in immune cell residency across the age range may be partially caused by an increase in epithelial cell number without concurrent change in immune cell number. The mechanisms responsible for balancing the number of resident immune cells to epithelial cells in the NM are unclear. What these mechanisms are and how they might be altered across the age range require further exploration.

In addition, while our pooling strategy allowed us to obtain sufficient number of high-quality cells for cataloging all major cell populations it limited our ability for high-powered interindividual analysis of minor differences. Additionally, possible confounding factors including racial differences among our study populations, and the effect of non-SARS-CoV-2 respiratory viruses could not be fully explored. However, we performed orthogonal and multi-dataset external validation whenever possible to try and identify if these factors created a major confounding effect.

In closing, our initial analyses of this resource show that the basic immunobiology of a tissue that is the primary site of infection for many viruses and bacteria of epidemiological concern, including SARS-CoV-2, is variable throughout the lifespan. This has implications ranging from our basic understanding of immune system development, and the process of aging, to more applied areas of study such as vaccination recommendations and patient treatment strategies in disease. Additionally, these results highlight the importance of studying biological processes in varying populations specifically across the age range.

## Methods

### Patient recruitment

All study subjects were enrolled at Children’s Mercy using salvage sample collection protocol (IRB # STUDY00001258). Both the discovery and replication NM cohorts of COVID-19 negative individuals included (1) patients aged 0 to 18 years tested for SARS-CoV-2 as part of their standard of care procedure, and (2) healthcare workers tested for SARS-CoV-2 as part of employee screening procedure at Children’s Mercy, respectively.

### Mid-turbinate swab sample collection

All individuals were tested for SARS-CoV-2 by mid-turbinate swabs using either Pediatric Copan FLOQSwabs (Copan Cat No. 56780CS01) or Adult FLOQSwabs (Copan Cat No. 56380CS01). Respiratory samples were stored at 4 °C in BD Universal Transport Media (BD Cat No. 220220) until transported to the clinical microbiology laboratory for SARS-CoV-2 testing. Experimental sample processing began within 12 h of sample collection after confirmation of negative test for SARS-CoV-2 by RT-PCR.

### Cell collection from mid-turbinate swabs and cryopreservation

Each sample was diluted with cold PBS (Thermo Fisher Cat No. 14190144) + 2% FBS (GE Healthcare Cat No. SH30088.03HI) up to a total volume of 5 mL and passed through a 40-µm nylon mesh cell strainer that had been prewetted with 2 mL of PBS + 2% FBS. The strainer was then rinsed with 7 mL of cold PBS + 2% FBS. The sample was transferred to a 15-mL conical tube and centrifuged at 300 × *g* at 4 °C for 8 min. The supernatant was carefully removed without disturbing the cell pellet. The cell pellet was resuspended in 200 µL of cold PBS + 2% FBS, and the cell count and viability were assessed using 0.4% Trypan Blue and a Countess II automated cell counter. The cell suspension was transferred to a 1.5-mL tube and centrifuged at 300 × *g* at 4 °C for 8 min, and the supernatant was carefully removed without disturbing the cell pellet. The cell pellet was resuspended in 1 mL of cold Recovery Cell Culture Freezing Medium (Thermo Fisher Cat No. 12648010), and the cell suspension was transferred to a cryogenic storage vial. The cryogenic storage vial was placed in a Corning CoolCell FTS30, which was then placed in a − 80 °C freezer overnight. Samples were stored at − 80 °C for no longer than one week before being thawed and processed for scRNAseq.

### Cell pooling and single-cell RNA- sequencing of NM

Samples with less than 30% viability were excluded from analysis and cells were used in pools. We collected samples until we reached in total of 35 samples with at least 3 samples per age group. For the lifespan single-cell atlas, six groups were formed based on the age of the patients with the youngest and the oldest pool being as extreme as possible (average age 9 months vs 50 years of age) based on the demographic of the study population (patients vs. health care workers) and the remaining groups with no more than 5 years age difference as follows: Control Group 1) 4 months–18 months (n = 7); Control Group 2) 5 years–9 years (n = 5); Control Group 3) 11 years–15 years (n = 6); Control Group 4) 16 years–19 years (n = 9); Control Group 5) 30 years–35 years (n = 4); and Control Group 6) 36 + years (n = 4). To reduce cell stress caused by delays due to processing multiple samples in parallel, the 6 lifespan pools were processed in 2 batches of 3 groups each. Samples within the first batch were thawed and pooled as described below and carried through to the first stable pause point in the scRNAseq protocol (GEM-RT Incubation); the process was then repeated for the second batch of samples. Once both batches of samples had reached the first pause point, all samples were processed in parallel to completion. We further processed an additional pool as replication consisting of three nasal samples derived from infants age 3 weeks–8 months (average age 4 months).

For each sample to be thawed, 10 mL of Thawing Medium consisting of DMEM/F-12 (Thermo Fisher Cat No. 11320033) supplemented with 10% FBS and 100 units/mL of penicillin and 100 µg/mL of streptomycin (Thermo Fisher Cat No. 15140122) was prewarmed in a 37 °C bead bath. Each cryogenic storage vial containing a sample to be thawed was placed in the 37 °C bead bath. No more than 5 samples were thawed at a time. When only a small ice crystal remained in the sample, both the cryogenic storage vial and the 15-mL conical tube containing the Thawing Medium were aseptically transferred to the biosafety cabinet. 1 mL of Thawing Medium was slowly added, dropwise, to the sample. The diluted sample was then mixed gently by pipetting and further diluted in the remaining 9 mL of Thawing Medium. The thawed and diluted cells were left at room temperature while the remaining samples were similarly thawed. When all samples in the batch were thawed, the samples were centrifuged at 300 × *g* for 8 min. The supernatant was carefully removed without disturbing the cell pellets. The cell pellets were each resuspended in 0.5 mL of Thawing Medium, and the cell suspensions were placed on ice. The cell suspensions were then combined and pooled together in age-defined groups. Each resulting pool was passed through a prewetted 40-µm nylon mesh cell strainer, and the cell strainers were rinsed with 5 mL of cold Thawing Medium. The pooled cell suspensions were centrifuged at 300 × *g* for 8 min at 4 °C, and the supernatant was carefully aspirated without disturbing the cell pellets. The cell pellets were resuspended in 100 µL of cold Thawing Medium, and cell count and viability were assessed using 0.4% Trypan Blue and a Countess II automated cell counter. For each group, 4 wells of a Chromium Chip B (10 × Genomics Cat No. 1000153) were loaded with 20,000 cells each. Following cell loading, scRNAseq was performed identically for all samples using the Chromium Single Cell 3’ Library & Gel Bead Kit v3 (10 × Genomics Cat No. 1000075) according to the manufacturer’s protocol.

### Specimen pooling and DNA isolation for epigenome analysis

Nasal specimens (N = 35) were stored at − 80 °C and were brought to room temperature before pooling by age. Before pooling, the specimens were mixed well with gentle pipetting. 100µL from each specimen was removed and pooled together in a 1.5 mL tube. Once all specimen aliquots were added to the pool, the pool was mixed by pipetting and 200 μl was taken from each pool into a new 1.5 mL tube for DNA isolation. DNA was isolated with a DNeasy Blood and Tissue Kit (Qiagen, Cat No. 69504) with the following modifications to kit protocol: 8uL of RNase A was used instead of 4ul during the optional RNase A step and the lysis incubation time at 56 °C was increased to at least 3 h to ensure complete lysis of the specimens. After isolation, the DNA concentration of each sample was determined using a Qubit dsDNA HS Assay Kit (Fisher, Cat No. Q32851).

### WGBS library preparation

100 ng of DNA was aliquoted from each sample. Unmethylated λDNA was added to each sample at 0.5%w/v and the samples were sheared mechanically using a Covaris LE220-plus system to a length of 350 bp, using the settings recommended by the manufacturer. The sizing was determined by a High Sensitivity D1000 ScreenTape and Reagents (Agilent, Cat. No. 5067-5584 and 5067-5585) on the TapeStation platform. Once the input DNA was at the proper fragment size, the samples were concentrated with a SpeedVac to a volume of 20µL. The samples then underwent bisulfite conversion with an EZ DNA Methylation- Gold kit (Zymo, Cat. No. D5006). The samples were eluted off the spin columns with 15 μl of low EDTA TE buffer (Swift, Cat. No. 30024) before library preparation.

The low-input libraries were prepared using an ACCEL-NGS Methyl-Seq Library kit (Swift, Cat. No. 30024) with a Methl-Seq Set A Indexing Kit (Swift, Cat. No. 36024), following the protocol associated with the library kit. During the protocol, bead cleanup steps were performed with SPRIselect beads (Beckman Coulter, Cat. No. B23318). Following the recommendation of the kit, 6 PCR cycles were performed to amplify the samples. The final libraries were quantified with a Qubit dsDNA HS Assay Kit and the size was determined by using a BioAnalyzer High Sensitivity DNA Kit (Agilent, Cat. No. 5067-4626).

### Sequencing

Sequencing was performed using an Illumina NovaSeq 6000. Runs of WGBS were 2 × 151 cycle paired-end, while runs of scRNAseq were 2 × 94 cycle paired-end.

### Post-sequencing analysis scRNAseq

Sequenced reads were initially processed by the cellranger pipeline (v3.1.0) which includes fastq creation, read alignment, gene counting, and cell calling. All samples were mapped to the cellranger GRCh38 v1.2.0 genome. The resulting cell by gene matrix from the cellranger “count” step was then processed using standard workflows in Seurat^29,30^. In brief, low quality cells were removed by filtering out cells with a unique gene count lower than 750 and more than 50% mitochondrial reads. The gene counts for remaining cells that passed quality control were then normalized using SCTransform^31^ with the replicate captures as a batch variable. For the age-range comparisons, the 6 age pools were integrated using the FindIntegrationAnchors and IntegratedData functions in Seurat with default parameters. The integrated data was then used for linear and non-dimensional reduction, nearest neighbor finding, and unsupervised clustering. Cell types were assigned by examining expression of known genes in the unsupervised clusters, as well as examining markers of the clusters identified using the FindAllMarkers function in Seurat with default parameters.

### Post sequencing analysis WGBS

Sequenced reads were initially processed by the DRAGEN (Edico/Illumina) pipeline (v.1.1.5). Following DRAGEN alignment, duplicate reads were marked with Picard tools^32^ MarkDuplicates and subsequently removed. Bismark^33^ was used to assign the methylation ratio at each CpG site. CpG sites were filtered to remove sites that had less than 10 × coverage, overlapped ENCODE problematic regions, or overlapped sites in dbSNP as previously described^34^.

### Genome-wide age correlated expression in immune cells

To identify pathways which were affected by age within immune cell types, we used the FindAllMarkers function implemented in Seurat to identify genes which were mostly highly expressed in each cell type. We selected genes which were at least log-twofold enriched in each cell type. For each cell type we performed a linear regression of age by expression of each marker gene using the lm function implemented in R.

### Methylation profile analysis

The immune cell composition of the NM was inferred from the methylation profile of samples that were independent of the single-cell RNA-sequencing samples. We measured CpG methylation in regions that have been identified to be primarily accessible in lymphoid and myeloid cells through large scale DNase 1 hypersensitive sequencing and analysis^17^. The regions that were identified as invariantly accessible across cell types were used as a control to test for overall methylation differences. We compared average methylation of CpGs in these regions across the age range and tested for overall shifts in the distributions with a Kruskal–Wallis test. In each case, the result was significant and all pairwise comparisons were made using a Mann–Whitney U test.

### Sex related immune residence analysis

Using the donor origin assignments obtained from Vireo^8^, we examined the expression of the X-inactivation transcript XIST in the cells of each identified donor. The number of individuals expressing this marker at a high level in each age pooled matched the known number of females in the pool and therefore was used as a proxy for donor sex.

### Cell-type composition comparison of adult samples

The cellular composition of adult NM samples was compared to with that of healthy age-matched controls from independent data^16^ by filtering cells with identical QC metrics, scaling both datasets independently with SCTransform and integrating the data using standard Seurat methods. The resulting clusters were then assigned a cell type by manual curation of marker genes. Finally, cellular composition of the samples was calculated and compared.

### Statistics

Differences in expression values between groups of cells were determined using the FindMarkers function in Seurat with default parameters.

### Oversight and ethics

All experimental protocols were determined as non-human subjects research by The Office of Research Integrity at Children’s Mercy Research Institute (# STUDY00001258) as only collection of existing de-identified specimens were included with no codes or linkers of any sort are maintained or available to the research team that would permit access to PHI or information about the living individual. As such, an Institutional Review Board (IRB) review was waived as this is needed only for studies that engage Children’s Mercy in Human Subjects Research.

## Supplementary Information

Supplementary Information 1.

Supplementary Information 2.

## Data Availability

Raw and processed data from all pooled samples is available via the Gene Expression Omnibus (scRNAseq accession number: GSE162864, WGBS accession number: GSE168254). Fully processed data are available for exploration through the UCSC cell browser (lifespan-nasal-atlas.cells.ucsc.edu).
