# Supplementary material for: Immune cell residency in the nasal mucosa may partially explain respiratory disease severity across the age range
Source: Sci Rep. 2021 Aug 5;11:15927. doi: 10.1038/s41598-021-95532-3 (PMC8342554; doi:10.1038/s41598-021-95532-3)
Supplement: Supplementary file 1 — Supplementary Information 1. [file 41598_2021_95532_MOESM1_ESM.pdf]

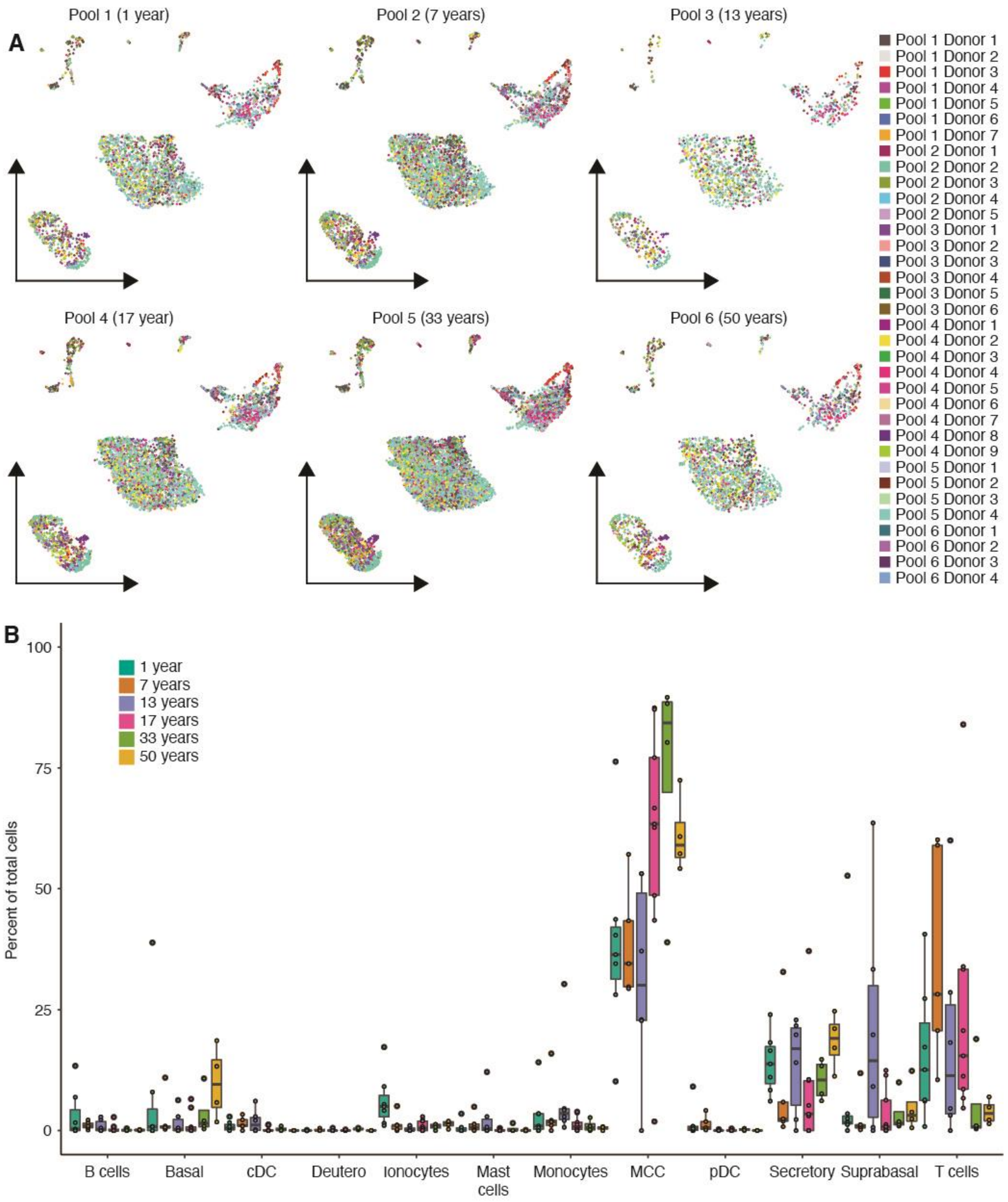

Supplemental Figure 1: NM cell type distributions are consistent across individuals within an age pool. A) UMAP projection of NM datasets split by each age pool and colored by the donor of origin within each pool. UMAP projection is identical to figure 1A. B) Quantification of A.

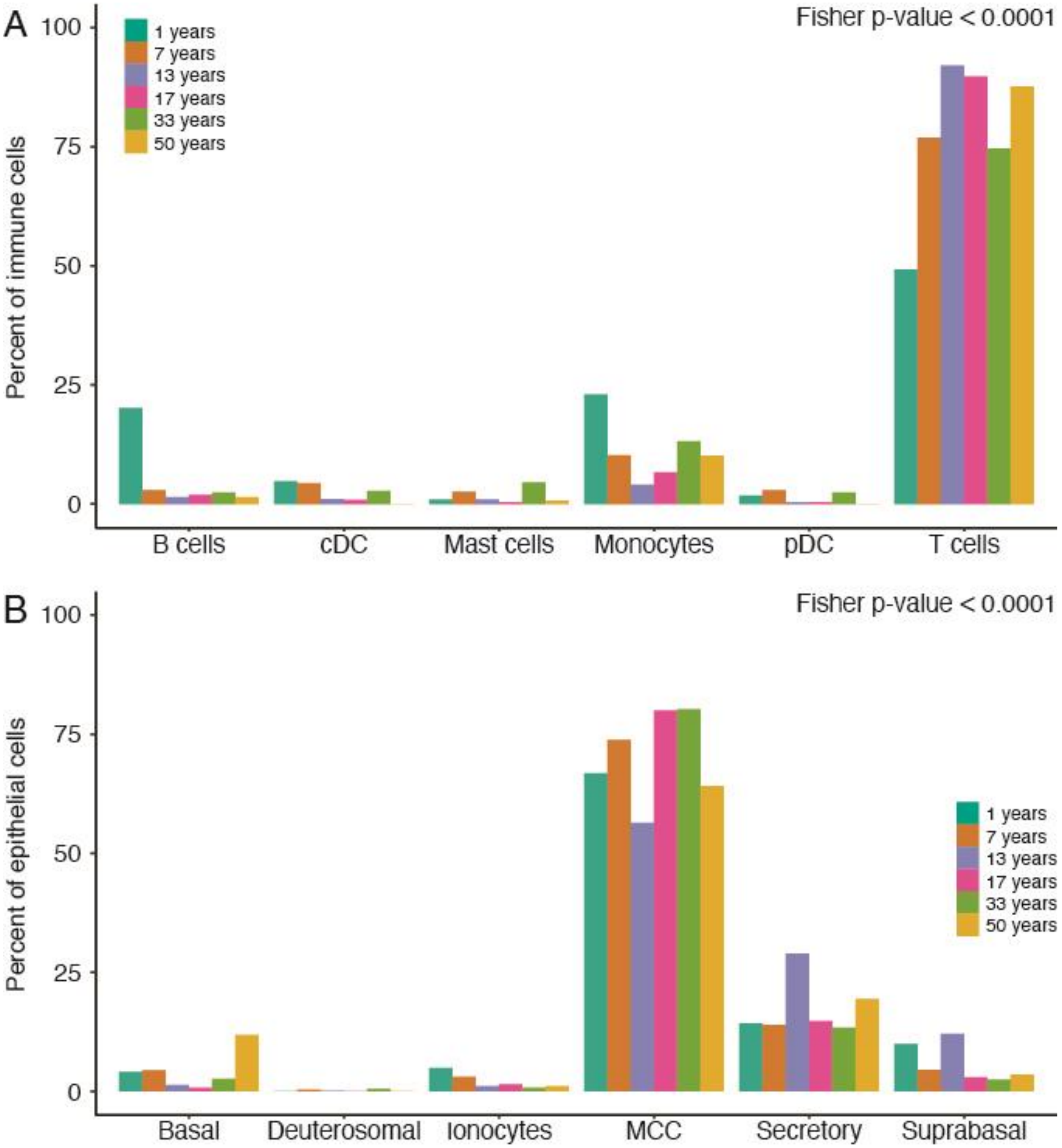

Supplemental Figure 2: Some innate immune cell types decrease in proportion with age. A-B) Percent of cells in the immune (A) and epithelial (B) cell subsets from Figure 1C that fall into each cell type.

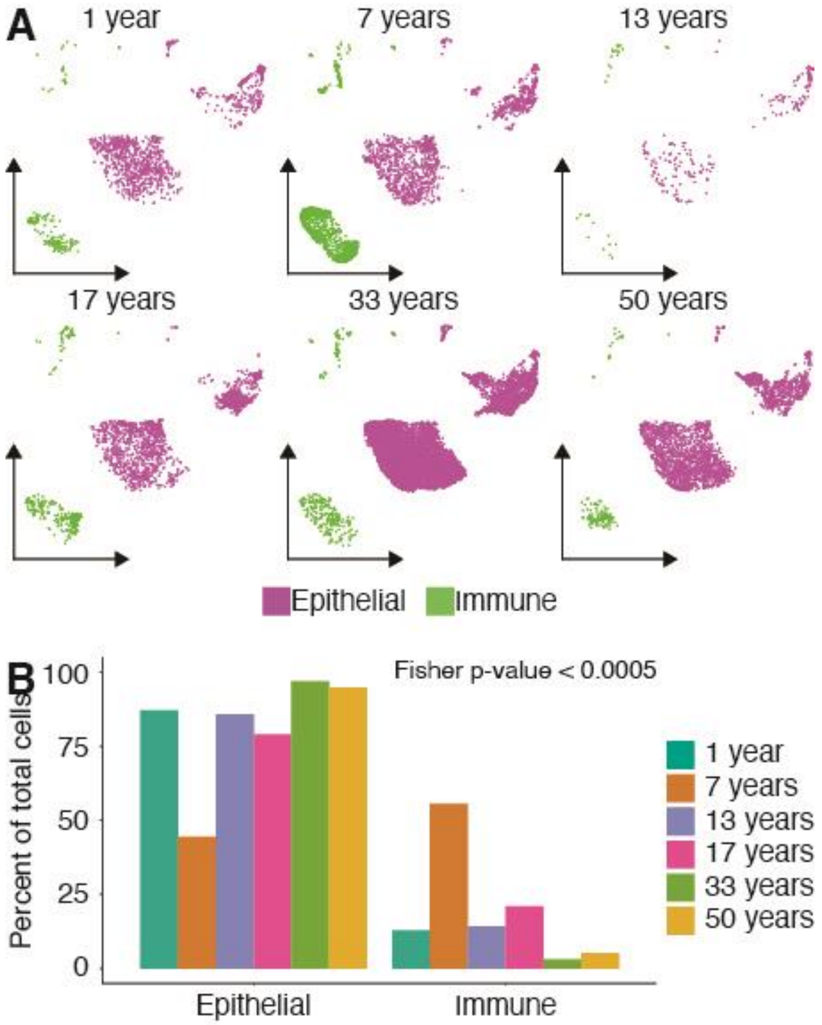

Supplemental Figure 3: Sex related effects are not part of immune cell residency trend. A-B) Same as figure 1 B-C except restricted to female donors only.

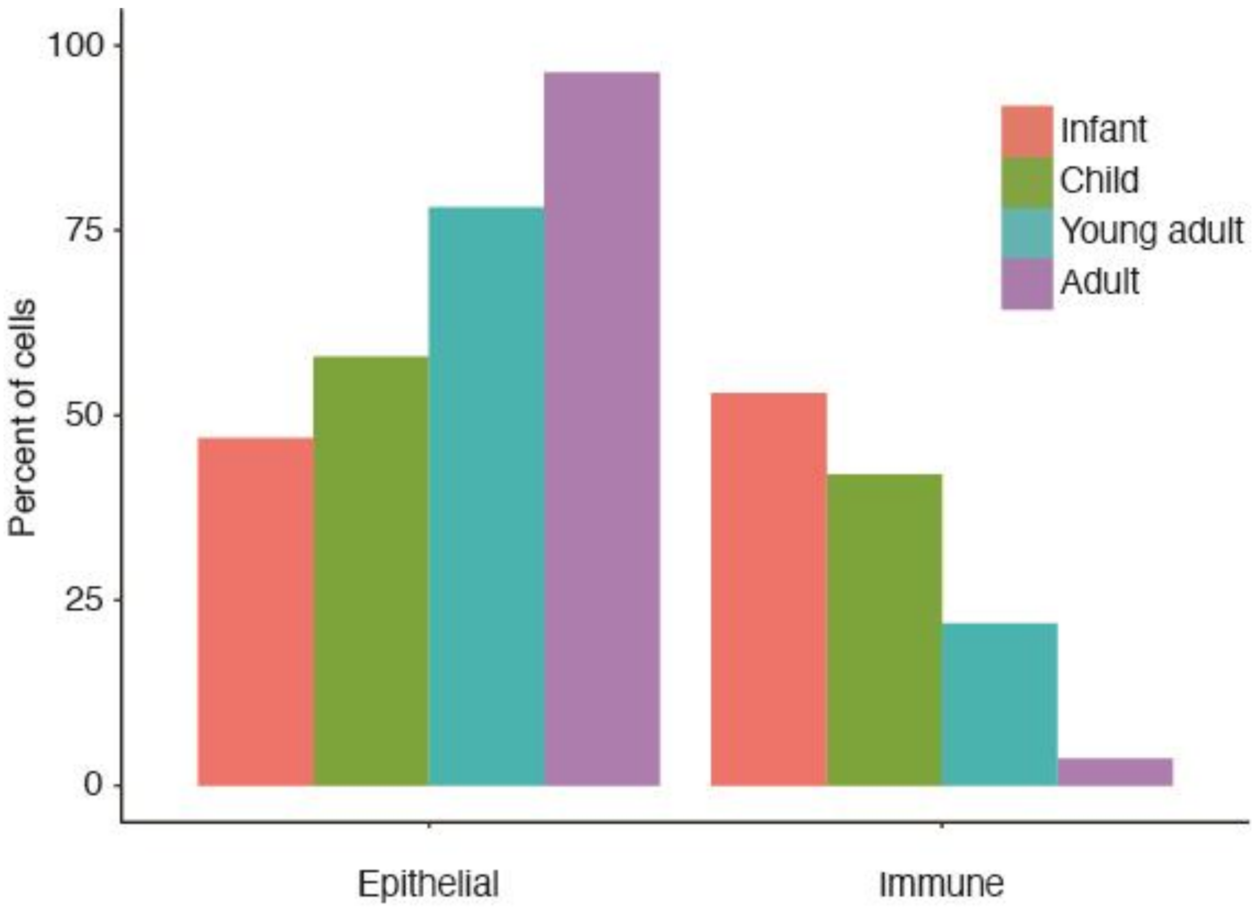

Supplemental Figure 4: Replication cohort of NM scRNAseq exhibits age-related immune cell residency. Infant data is comprised of replication cohort. The discovery cohort has been grouped into “Child”(Discovery Pools 1-3), “Young Adult” (Discovery Pool 4), and “Adult” (Discovery Pools 5-6) groups for this comparison.

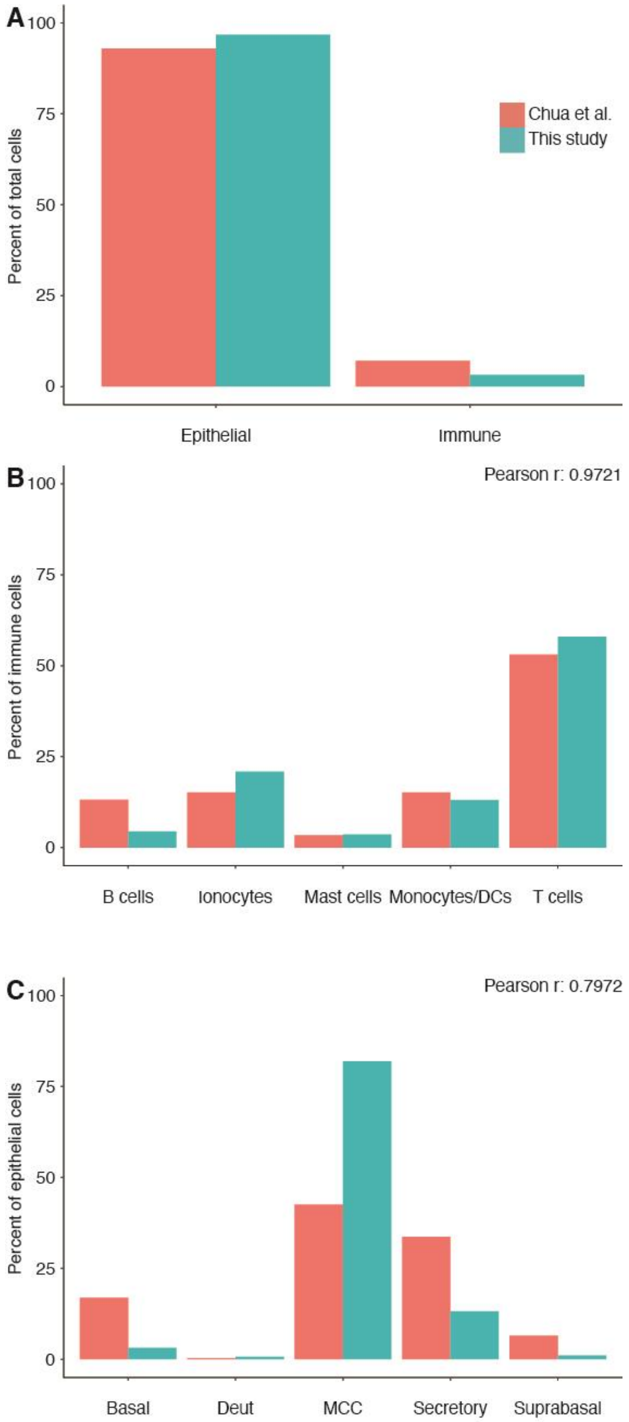

Supplemental Figure 5: Age-matched cell type proportions in the NM are consistent with other datasets. A) Comparison of epithelial and immune cell proportions in the NM of adults identified by scRNAseq. B-C) Percent of cells in the immune (B) and epithelial (C) cell subsets that fall into each cell type. Average pool age in years: “This study” – 33.3, “Chua et al.” – 35.85.

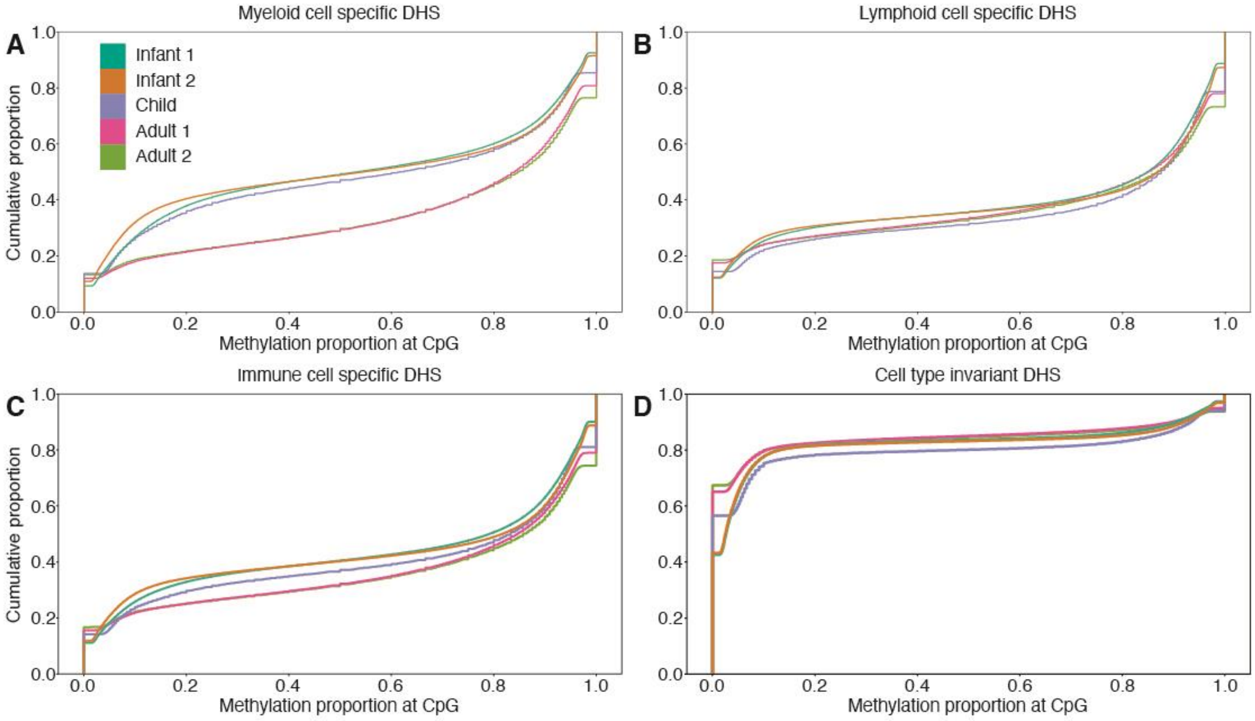

E

Group 1	Group 2	Myeloid p-value	Lymphoid p-value	Immune p-value	Invariant p-value
Infant 1	Infant 2	< 0.0001	> 0.999	> 0.999	< 0.0001
Infant 1	Child	< 0.0001	< 0.0001	< 0.0001	> 0.999
Infant 1	Adult 1	< 0.0001	< 0.0001	< 0.0001	> 0.999
Infant 1	Adult 2	< 0.0001	< 0.0001	< 0.0001	> 0.999
Infant 2	Child	< 0.0001	< 0.0001	< 0.0001	> 0.999
Infant 2	Adult 1	< 0.0001	< 0.0001	< 0.0001	> 0.999
Infant 2	Adult 2	< 0.0001	< 0.0001	< 0.0001	> 0.999
Child	Adult 1	< 0.0001	> 0.999	< 0.0001	> 0.999
Child	Adult 2	< 0.0001	< 0.0001	< 0.0001	> 0.999
Adult 1	Adult 2	< 0.0001	< 0.0001	< 0.0001	> 0.999

Supplemental Figure 6: Epigenomic analyses supports the hypothesis that immune residence in the NM decreases with age. A-D) Empirical cumulative distribution function of CpG methylation for all CpGs that overlap DHS sites identified as being enriched in (A) myeloid cells, (B) lymphoid cells, (C) immune (myeloid + lymphoid) cells, and (D) DHS sites that are invariant across cell types. E) Mann-Whitney rank test p-value for the hypothesis that the median methylation ratio of CpG sites overlapping the specified DHS peaks of Group 1 is less than that of Group 2. Average pool age in years: Infant 1 – 0.183, Infant 2 – 0.55, Adolescent – 5.275, Adult 1 – 29.125, Adult 2 – 40.57.

Supplemental Table 1: Human subject information and pooling strategy (Discovery Cohort)

Supplemental Table 2: Cell type marker genes

Supplemental Table 3: Human subject information and pooling strategy (Replication Cohort)

Supplemental Table 4: Immune genes analyzed via linear models

Supplemental Table 5: Total cell counts
